# Molecular Mechanisms of Nickel Allergy

**DOI:** 10.3390/ijms17020202

**Published:** 2016-02-02

**Authors:** Masako Saito, Rieko Arakaki, Akiko Yamada, Takaaki Tsunematsu, Yasusei Kudo, Naozumi Ishimaru

**Affiliations:** Department of Oral Molecular Pathology, Institute of Biomedical Sciences, Tokushima University Graduate School, 3-18-15 Kuramoto Tokushima 770-8504, Japan; m.saito@tokushima-u.ac.jp (M.S.); arakaki.r@tokushima-u.ac.jp (R.A.); aki.yamada@tokushima-u.ac.jp (A.Y.); tsunematsu@tokushima-u.ac.jp (T.T.); yasusei@tokushima-u.ac.jp (Y.K.)

**Keywords:** metal allergy, Ni, DTH, DC, T cell, TLR, TSLP

## Abstract

Allergic contact hypersensitivity to metals is a delayed-type allergy. Although various metals are known to produce an allergic reaction, nickel is the most frequent cause of metal allergy. Researchers have attempted to elucidate the mechanisms of metal allergy using animal models and human patients. Here, the immunological and molecular mechanisms of metal allergy are described based on the findings of previous studies, including those that were recently published. In addition, the adsorption and excretion of various metals, in particular nickel, is discussed to further understand the pathogenesis of metal allergy.

## 1. Introduction

Contact dermatitis is usually caused by external exposure of the skin to allergens, such as metals, chemicals, and plants. Metal allergy is an inflammatory disease categorized as a delayed-type hypersensitivity (DTH) reaction. Humans come in contact with various metals daily. For example, metal alloys are widely used in costume jewelry, dental materials, or glasses. Although many individuals develop a metal allergy, the precise molecular mechanism underlying this allergy remains unknown.

Some metals cause contact allergic reactions categorized as type IV DTH, in which skin inflammation is mediated by hapten-specific T cells [1,2]. In this review, the cellular and molecular mechanisms identified by basic and clinical studies on metal allergy are described. In addition, the adsorption and excretion of metals in the human body and useful animal models for investigating metal allergy are reviewed. Furthermore, the adsorption and excretion of metals in the body are discussed. Finally, the pathogenesis of metal allergy is described with respect to the potential molecular mechanisms of this immune response.

## 2. Metal Allergy

Metals, such as gold (Au), silver (Ag), mercury (Hg), nickel (Ni), titanium (Ti), chromium (Cr), copper (Cu), and cobalt (Co) are ubiquitous in our environment and are widely used in costume jewelry, coins, mobile phones, and dental materials. Approximately, 10%–15% of the human population suffers from contact hypersensitivity to metals [1,2]. This allergy is considerably more common in women than in men, with an approximate population frequency of 10% in women *vs.* 2% in men [3,4]. Clinically, metal allergy is related to the cause of contact dermatitis, pustulosis palmoplantaris, lichen planus, dyshidrotic eczema, and burning mouth syndrome [5,6,7,8]. Moreover, patients with autoimmune conditions, including systemic lupus erythematosus, rheumatoid arthritis, and Sjögren’s syndrome, have an increased frequency of metal allergy [9].

A previous study indicated that nickel (II) sulfate has the highest sensitization rate and affects approximately 15% of the population, followed by cobalt chloride and potassium dichromate, which approximately 5% and 3% of the population, respectively [10]. Nickel allergy is the most common [2,11], and clinically important condition that is becoming a threat to public health [12,13]. The use of nickel alloys is common in dentistry, and high concentrations of nickel can be found in food. Nickel-casting alloys are cheap and have favorable physical properties but are prone to corrosion in the oral environment [14]. Metal allergy is mainly diagnosed by patch testing. Several reports have demonstrated that the removal of causal metal can successfully improve allergic symptoms. Therefore, in addition to the metal concentration, a special quality of metal seems to be important for the pathogenesis of metal allergy [15,16,17].

Nickel ions released from various alloys are potent allergens or haptens that can trigger skin inflammation [18,19,20]. They penetrate the skin and activate epithelial cells that produce various cytokines or chemokines. The reaction follows complex immune responses that involve the activation of antigen-presenting cells (APCs) and T cells [21,22,23]. Some cytokines activate APCs, such as Langerhans cells (LCs) or dendritic cells (DCs). Activated APCs migrate to the draining lymph nodes where they present the allergens or haptens to naive CD4-positive T cells. Subsequent re-exposure to the same allergen or hapten would lead to the activation of hapten-specific T-cells, which subsequently enter the bloodstream and produce visible signs of hypersensitivity at 48 to 72 h after allergen or hapten exposure [24]. However, the precise molecular mechanisms that mediate the interactions between epithelial and immune cells in nickel allergy remain unknown.

## 3. Animal Models and Molecular Mechanism of Metal Allergy

Many researchers have used animal models to investigate nickel allergy by administrating adjuvants. For example, nickel chloride (II) is administered twice into mice in combination with adjuvants, such as incomplete Freund’s adjuvant and complete Freund’s adjuvant and ear swelling is evaluated for DTH after 48 h [25].

### 3.1. Keratinocytes and APCs in Ni Allergy Models

**Figure 1:**
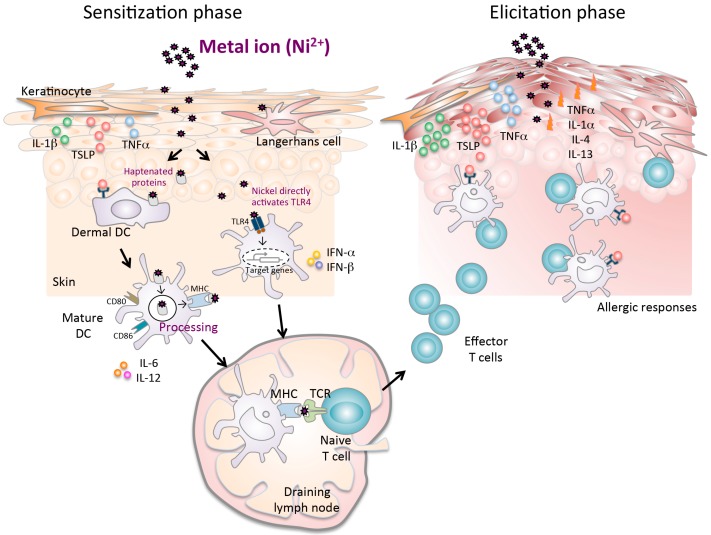
A complex mechanism of metal allergy. The sensitization phase begins after nickel exposure to the skin. Nickel penetration into the skin results in the production of proinflammatory cytokines (TNF-α and IL-1β), TSLP, and chemokines, which induce activation and migration of haptenated protein-loaded epidermal and dermal DCs through afferent lymph to the draining lymph nodes. Particularly in humans, nickel directly activates the TLR4 pathway in DCs. In the draining lymph nodes, haptenated-peptide presentation results in the proliferation, activation and subsequent differentiation of hapten-specific T cells. Secretion of cytokines in the draining lymph nodes during the sensitization phase contributes to efficient hapten-specific T cell activation, proliferation, and differentiation. At the end of this phase, primed specific T cells migrate out of the lymph nodes to the skin. In the elicitation phase, the subsequent application of the same hapten leads to uptake by cells, which is presented to the recirculating hapten-specific T cells. The activated T cells produce inflammatory cytokines and chemokines at the site of exposure that promote an allergic reaction, leading to the development of characteristic skin lesions.

Nickel penetrates the skin tissue and activates keratinocytes, leading to the release of certain cytokines such as interleukin (IL)-1β and tumor necrosis factor alpha. Subsequently, nickel attaches to the major histocompatibility complex (MHC) molecules on LCs and DCs that are upregulated by the cytokines from the surrounding keratinocytes. These cytokines control the expression of E-cadherin and chemokines, including matrix metalloproteinase-9, secondary lymphoid tissue chemokine (SLC), and macrophage inflammatory protein-3β, that are produced by the APCs [26,27,28,29]. Subsequently, the APCs migrate to draining lymph nodes where they present these haptens to naive T cells. Re-exposure to the same hapten induces a hypersensitive reaction in an effector phase at the site of exposure (Figure 1).

Several studies have shown that the activation of p38 mitogen-activated protein kinase in dermal DCs is required to trigger a T cell-mediated immune response in a mouse model of nickel allergy [30,31,32,33]. Ni-activated epithelial DCs or LCs exhibit the upregulation of CD80, CD83, CD86, and MHC class II [30]. Moreover, nickel plays an important role in the maturation and activation of immature LCs or DCs in the skin via phosphorylated MAP kinase kinase 6 (MKK6) [31,32,33,34]. Therefore, Ni-stimulated DCs prime activate T cells to induce skin inflammation at the site of exposure to nickel. However, the injection of short interfering (si) RNAs targeting *MKK6* prevents a hypersensitive reaction after Ni immunization in a mouse model, suggesting that manipulating *MKK6* in DCs might be a good therapeutic strategy for nickel allergy [25].

### 3.2. Critical Role of Toll-Like Receptor 4 in Ni Allergy

Human toll-like receptor (TLR) 4 has been shown to play a crucial role in the development of contact allergy to nickel [35]. TLR4-deficient mice expressing transgenic human TLR4 developed contact hypersensitivity to nickel, whereas those expressing mouse TLR4 did not [35]. Although the cell type contributing to a TLR4-mediated allergic reaction has not been identified, immune cells such as DCs, macrophages, and endothelial cells were found to be associated with the allergic reaction to Ni via TLR4 [35]. Ni-induced activation of TLR4 leads to the activation of nuclear factor (NF)-κB, p38, and interferon regulatory factor 3, resulting in the induction of multiple proinflammatory cytokines that trigger an allergic response. These findings explain why Ni^2+^, but not other contact allergens, directly triggers NF-κB-dependent activation of human DCs [35]. Furthermore, a recent study suggested that other metals, including cobalt and palladium, induce IL-8 production in HEK293 cells via TLR4/MD2 [36]. Lipopolysaccharides are an important inducer of nickel allergy and enhance the allergic response in TLR4-mutant mice [37]. However, TLR4 signaling by keratinocytes controls wound healing by inducing CCL5 expression [38]. Keratinocytes are known to produce danger signal-induced cytokines or chemokines in the skin tissue [39]. A recent study demonstrated that ionized gold is recognized by TLR3. Epithelial TLR3 plays a crucial role in the localized irritation reactivity to gold in the skin and mucosa. Therefore, in addition to gold, nickel, copper and mercury salts may activate an innate immune response in keratinocytes [40]. Taken together, these findings reveal a mechanism of skin contact allergy development and might contribute to the elucidation of novel therapeutic strategies such as those based on interference with distinct immune detention pathways.

### 3.3. Thymic Stromal Lymphopoietin and Its Receptor in Ni Allergy

Using a mouse model, a recent study showed that the increased expression of the thymic stromal lymphopoietin (TSLP) receptor (TSLPR) on DCs plays a key role in triggering an allergic response to nickel [41]. In this mouse model of nickel allergy, DCs in ear tissues were activated via TSLPR signaling induced by keratinocyte-derived TSLP. Furthermore, DTH reactions in mice with Ni-induced allergy were reduced significantly by the injection of a Tslp–siRNA combined with atelocollagen into the ear skin of the ear [41]. These results suggest that nickel allergy is triggered by a TSLP/TSLPR-mediated interaction between epithelial and immune cells. TSLP is produced by keratinocytes, the tonsil crypt epithelium, and bronchial epithelial cells [42,43]. Furthermore, TSLP induces allergic inflammatory reactions in patients with asthma and atopic dermatitis [42,43].

Although numerous patients develop allergic symptoms against various metals, experimental animal models of nickel allergy have been widely used to elucidate the molecular mechanisms of metal allergy. Allergic diseases are multifactorial disorders caused by various factors, such as genetics and the environment in addition to exposure to metals. Moreover, little is known regarding the molecular or cellular mechanisms underlying haptenization of metal allergens during an allergic reaction.

CD25^+^ T cells isolated from peripheral blood of human nickel-allergy patients demonstrated a limited or no capacity to suppress metal-specific CD4^+^ and CD8^+^ T cell responses. In contrast, CD4^+^CD25^+^ T cells from peripheral blood of non-allergic subjects strongly regulate immune responses to nickel in a cytokine-independent, cell-contact-dependent mechanism. These results indicate that in healthy individuals CD25^+^ Treg can control the activation of both naive and effector nickel-specific T cells [44,45].

Further studies on animal models might reveal the precise mechanism by which metal allergy promote the clinical application of new therapeutic strategies.

## 4. Adsorption and Excretion of Metals

Understanding how metals are metabolized in the body is one of the key factors in better elucidating the process of developing a metal allergy [46]. The accumulation of metals in the body is influenced by exposure time, absorption medium, tissue distribution, and metal excretion. Nickel has been well studied among the metals associated with allergy. The biological half-life of nickel is estimated to range from 17 to 39 h and 20 to 34 h in the urine and plasma, respectively [47]. The model allows the precise prediction of the state and extent of exposure, which is affected by varying concentrations of metals in the atmosphere [47].

The investigation using the excised human skin showed that Ni ions are detected to penetrate the skin using a very sensitive method to quantify the amount of nickel permeating to the skin [48,49,50]. Although the permeation process is slow with a lag time of approximately 50 h, the rate using aqueous nickel chloride is increased compared with that in aqueous nickel sulfate [50]. Thus, the selection of nickel salt is an important consideration when conducting a skin patch test for detecting nickel permeation to the skin [50].

Absorption of Ni via the gastrointestinal tract by diet remarkably affects the bioavailability of nickel in the body; approximately 25% of nickel ingested in drinking water after an over-night fast is absorbed from the intestine and excreted in the urine, whereas only 1% of nickel ingested is absorbed [51]. The compartmental model and kinetic parameters decrease the uncertainty of toxicological assessments of human exposures to Ni via drinking water and food [51].

The other well-studied example is cobalt. Water-soluble cobalt salts are rapidly absorbed from the small intestine, although the bioavailability of cobalt is limited and highly variable [52]. Cobalt uptake occurs substantially through the lungs following inhalation and cobalt oxide in dust, and welding fumes leads to the systemic dissemination of ultrafine particles via the lymph and vascular system, releasing soluble cobalt ions [52]. After a single dose of cobalt to humans, the concentration of cobalt in the serum is initially high but decreases rapidly by the tissue uptake, primarily by the liver and kidney combined with urinary and fecal excretion. Renal excretion is rapid but decreases over the first few days, followed by a slow phase lasting for several weeks. Therefore, the metal is sustained in the tissues for several years [53,54]. During the first 24 h, 40% cobalt is eliminated and approximately 70% is eliminated after a week. However, one month later approximately 20% and one year later approximately 10% remain [55].

The adsorption and excretion of metals in the human body are also controlled by genetic factors. A genome-wide association study (GWAS) demonstrated single nucleotide polymorphisms associated with whole blood levels of metals [56]. Eleven metals and trace elements including aluminum, cadmium, cobalt, copper, chromium, mercury, manganese, molybdenum, nickel, lead, and zinc, were evaluated in a cohort of 949 individuals by using mass spectrometry. In addition, DNA samples were also genotyped. This GWAS analysis revealed that two regions, 4q24 and 1q41, are associated with serum magnesium levels; these regions encode a protein involved in manganese and zinc transport, SLC39A8 and SLC30A10, respectively. These data revealed metabolic pathways of metals and suggested that different subsets of individuals are more susceptible to metal toxicity [56].

## 5. Conclusions and Perspectives

The incidence of allergic diseases has been increasing worldwide. The pathogenesis and mechanisms of the allergic response is highly complex, and many patients develop refractory disease. Because metal allergy is caused by materials used in products that are common in our daily life, chances of triggering the onset of allergic reactions are high. The clinical symptoms of metal allergy include rashes, swelling, and pain. Molecular pathogenesis of a metal allergy suggests that excess responses to metals occur via the complicated process of the interactions among the immune system, epithelial barrier, and homeostatic mechanism. The unique features, adsorption, and the excretion of metals in the human body complicate the pathogenesis and symptoms of metal allergy. Molecular mechanisms of metal allergy need to be determined to develop novel therapeutic strategies. Analysis and characterization of the precise mechanisms could have clinical implications leading to the development of new diagnostic or treatment methods for metal allergy.
